# Nanotechnology advances towards development of targeted-treatment for obesity

**DOI:** 10.1186/s12951-019-0554-3

**Published:** 2019-12-16

**Authors:** Nicole Remaliah Samantha Sibuyi, Koena Leah Moabelo, Mervin Meyer, Martin Opiyo Onani, Admire Dube, Abram Madimabe Madiehe

**Affiliations:** 1 Department of Science and Technology/Mintek Nanotechnology Innovation Centre, (DST/Mintek NIC), Biolabels Node, Department of Biotechnology, University of the Western Cape (UWC), Bellville, 7535 South Africa; 2 Nanobiotechnology Research Group, Department of Biotechnology, UWC, Bellville, 7535 South Africa; 3 Organometallics and Nanomaterials, Department of Chemistry, UWC, Bellville, 7535 South Africa; 4 Infectious Disease Nanomedicine Research Group, School of Pharmacy, UWC, Bellville, 7535 South Africa

**Keywords:** Angiogenesis, Nanotechnology, Obesity, Targeted delivery, White adipose tissue

## Abstract

Obesity through its association with type 2 diabetes (T2D), cancer and cardiovascular diseases (CVDs), poses a serious health threat, as these diseases contribute to high mortality rates. Pharmacotherapy alone or in combination with either lifestyle modification or surgery, is reliable in maintaining a healthy body weight, and preventing progression to obesity-induced diseases. However, the anti-obesity drugs are limited by non-specificity and unsustainable weight loss effects. As such, novel and improved approaches for treatment of obesity are urgently needed. Nanotechnology-based therapies are investigated as an alternative strategy that can treat obesity and be able to overcome the drawbacks associated with conventional therapies. The review presents three nanotechnology-based anti-obesity strategies that target the white adipose tissues (WATs) and its vasculature for the reversal of obesity. These include inhibition of angiogenesis in the WATs, transformation of WATs to brown adipose tissues (BATs), and photothermal lipolysis of WATs. Compared to conventional therapy, the targeted-nanosystems have high tolerability, reduced side effects, and enhanced efficacy. These effects are reproducible using various nanocarriers (liposomes, polymeric and gold nanoparticles), thus providing a proof of concept that targeted nanotherapy can be a feasible strategy that can combat obesity and prevent its comorbidities.

## Introduction

Obesity is a global epidemic that results from increased energy intake and storage of excess fat within the WAT depots [1–3]. Prolonged energy imbalance due to excess energy intake and less energy expenditure has been associated with development of life threatening diseases such as CVDs, some form of cancers and metabolic diseases [4, 5]. As a chronic disease, anti-obesity treatments relies on lifestyle modification, surgery, and pharmacotherapy. Pharmacotherapy in combination with lifestyle modification and surgery, is used to maintain a healthy body weight [5]. However, the success of anti-obesity drugs in reducing body weight is often counteracted by side effects, which have led to withdrawal of several anti-obesity drugs from the market [1, 2, 5]. There is clearly a need for new and improved anti-obesity strategies with sustainable weight loss effects and minimal bystander effects towards healthy tissues. Dysfunction in the WATs is the root cause of obesity and obesity-related diseases, hence this tissue is an ideal target for therapeutic intervention. The WATs express more than fifty adipokines, some of which are implicated in the development of obesity and its associated metabolic diseases [6–8]. Leptin [7, 9] and adiponectin [7, 10], expressed primarily by the adipocytes, are on top of the list. Prohibitin (PHB) [11] and integrin [12] are used as WAT vascular biomarkers in pre-clinical studies, and have shown potential for clinical application. Therefore, the WATs can be exploited through various molecules secreted or expressed by the cells that make up this tissue (adipocytes, lymphocytes, macrophages, fibroblasts, and vascular cells) for treatment of obesity.

Targeting the disease-associated biomarkers might provide insights on the pathophysiology, prevention and management of obesity and its related diseases [8], and could potentially be used for improving the efficacy of existing drugs or those that had been withdrawn from the market. Great efforts have been made through targeted nanotechnology-based treatments, where nanocarriers were used as drug delivery vehicles to improve the pharmacokinetics of anti-obesity agents. This review focuses on three potential nanotechnology-based anti-obesity strategies targeted on the WAT and its vascular system. These treatment strategies are based on the following three mechanisms (1) inhibition of angiogenesis in the WAT vasculature [13–15], (2) transformation of WAT into BAT [12, 16, 17], as well as (3) photothermal lipolysis of WAT [18, 19]. The targeted nano-based systems were able to deliver the therapeutic agents specifically at the local tissues, reduce drug toxicity towards healthy tissues, and was more effective at very low dosages [13–19]. The preclinical studies presented in this review article provide evidence that targeted nanotherapy could serve as an alternative treatment for obesity and prevent its progression to obesity-induced diseases.

## Obesity and challenges of current drug treatments

Obesity is a global health concern and a chronic disease that affects millions of people worldwide, both children and adults alike [5, 20]. The epidemic has been growing steadily long before it was considered to be a disease by the American Medical Association in 2013 [21]. The global statistics for adult males and females (≥ 18 years) indicate that since 1975 women are more prone to obesity than men. Of concern is that these rates have more than tripled for men, from 3.2% in 1975, which was half of what was recorded for women (6.4%) to 11% in 2016 which is 4% less than the rate for women. The current global statistics indicate that 13% (650 million) of the adult population are obese, while 1.9 billion are overweight [20]. These numbers are alarming, and are expected to escalate if not addressed. It is estimated that 60% of global deaths in the next 6 years (2025) will be caused by obesity-related diseases [5, 20]. These projections highlight the need for the obesity epidemic to be addressed with more urgency [20].

Obesity occurs due to energy imbalance between energy intake and energy expenditure in the WATs for a prolonged period. In genetically susceptible individuals, it can be triggered by environmental factors such as high caloric diets and lack of physical activity. Body mass index (BMI), i.e. BMI = weight (kg)/height^2^ (m) is the most widely used method for measurement of obesity. Generally, individuals with a BMI range of 18.5–24.9 kg/m^2^ are considered to be normal (healthy), while those with a BMI that is < 18.5 and ≥ 25 kg/m^2^ are said to have unhealthy body weights. According to the BMI weight status, adults (≥ 18 years) are categorized as overweight at a BMI ≥ 25–29.9 kg/m^2^, and anyone with a BMI ≥ 30 kg/m^2^ are classified as obese [1–3, 22]. If left untreated for a longer period, obesity can predispose patients to various chronic diseases, which include metabolic, CVDs, inflammatory and malignant diseases [4, 5]. These diseases reduce life expectancy and contribute to high mortality rates, of which cancer and CVDs are among the leading causes of death worldwide. Obesity is a chronic disease and currently there is no single treatment strategy that can completely cure it. Clinically it can be managed through lifestyle modification, pharmacotherapy, and surgery in severe cases [1, 2, 5].

Obesity management primarily relies on pharmaceutical drugs to maintain a healthy body weight and prevent its progression to related chronic diseases [1, 23, 24]. These pharmaceutical drugs are only recommended for overweight and obese patients who are non-responsive to lifestyle modification within the first 6 months, and suffer from at least one of the obesity-induced diseases [1–3]. There are various anti-obesity drugs available for short-term and long-term use, these drugs work mainly in the central nervous system, gut and intestines to either suppress appetite, inhibit fat absorption, or increase energy expenditure [1, 2, 25]. Short-term drugs include: phentermine [26–28], diethylpropion, mazindol, benzphetamine, and phendimetrazine [25, 29]. Orlistat and liraglutide have been approved for long-term treatment of obesity [25, 30]. Orlistat reduces body weight by blocking fat absorption in the gut through inhibition of gastric and pancreatic lipases. However, the effectiveness of this drug have been overshadowed by side effects; limiting its use to a period not longer than 2 years [31, 32]. The side effects include oily spotting, flatus with discharge, fecal urgency, fatty/oily stool, oily evacuation, increased defecation, and fecal incontinence [26–28] Liraglutide is a human glucagon-like peptide-1 (GLP-1) receptor agonist. GLP-1 is an incretin hormone secreted by the L-cells of the gastrointestinal tract, the hormone stimulate insulin secretion, reduce blood glucagon levels; its anti-obesity activity are attributed to its ability to delay gastric emptying and appetite suppression effects. In addition to the drug ‘s gastrointestinal side effects, it increases the risk of pancreatitis and increases the heart rate [26–28, 33]. Furthermore, the drug is available as an injectable and not as a convenient oral dosage form, thus reducing patient’s compliance [33].

The usefulness of the anti-obesity drugs is mostly limited by non-specificity, poor efficacy, and bystander effects. In cases where the side effects surpassed the efficacy of the drugs, this led to the withdrawal of most potent drugs [23]. Some of the drugs that have been removed from the market include amphetamine analogs, sympathomimetics and cannabinoid agonists. The anti-obesity effects of these drugs (e.g. aminorex, ephedrine, fenfluramine-phentermine, rimonabant, and sibutramine) were tainted by their undesirable toxic side effects which included cardiac valve defects, pulmonary hypertension, stroke, psychotic behaviors, addiction liability, and in some cases death [1, 2, 24, 25].

For the recent FDA-approved anti-obesity drugs, failure to lose at least 5% of the body weight within 12 weeks calls for drug discontinuation [34]. To date there is no pharmacologic therapy that has succeeded in maintaining a weight loss of over 10% for 1 year. For some, bariatric surgery (e.g. gastric bypass) is an option, however, weight regain is a common occurrence following surgery. Thus, there is an urgent need to find or develop alternative treatment strategies with minimal side effects and sustainable weight loss effects. This can be achieved by targeting the tissues that are directly involved in obesity development, mainly the WATs, targeted therapy might be more effective in treating obesity as a disease. Treating the underlying disease will in turn reduce the obesity-induced chronic diseases.

### AT as an ideal target for obesity treatment

Formerly thought to be an inert fat storage organ, the adipose tissue (AT) has been recognized as an endocrine organ that plays a crucial role in the body’s homeostasis [35–37]. It accounts for less than 30 and up to 80% of total body weight mass in lean and obese humans, respectively. There are two main types of ATs, that differ in their function and anatomical location, namely, the brown adipose tissue (BAT) and the white adipose tissue (WAT) [35, 36, 38]. The BAT is located mainly in the intrascapular or supraclavicular region [23, 39, 40]. Intrascapular BAT is abundant in rodents and hibernating animals. In humans, BAT is more prominent in newborn babies and adults with high metabolic rates [23, 39–41]. Human BAT function is high in newborns, and diminishes with age and increased body weight. The energy expenditure in BAT is promoted by high levels of uncoupling protein (UCP)-1 in brown adipocytes. BAT is an energy dissipating organ that is responsible for adaptive thermogenesis [38, 42–44]. It preserves homeostasis by uncoupling oxidative metabolism from ATP production in order to produce heat via UCP-1 during cold exposure. The recent identification of BAT in adult humans has sparked interest to explore it for obesity treatment [38, 45–48].

The WATs are found in various places all over the body. Their major depots include the subcutaneous (inguinal) which is located under the skin is the largest depot, and visceral (retroperitoneal, perirenal, epididymal, mesenteric, ovarian) depots, located around the kidney, intestines, pericardial, and ovaries. This tissue is mostly involved in storage of excess energy in the form of triacylglycerides (TAGs), which can be converted to glucose during the fasting state [36]. It is recognized that visceral WAT, which account for much less in body mass, poses a far greater risk for obesity patients. Up until 1994, the WAT was perceived as a passive organ that simply store excess fat. And after the discovery of leptin, it was evident this tissue, through its secretory molecules (known as adipokines), is involved in the metabolic and physiological functions of the body. Leptin, although secreted by the WAT, also regulates satiety signals in the brain [3, 9]. More than 50 other adipokines are known to date, which are responsible for the autocrine, endocrine and paracrine functions of the WAT. Some of their metabolic functions include glucose metabolism (adiponectin, resistin), lipid metabolism (adiponectin, TNF-α, interleukin-6), insulin sensitivity (adiponectin, visfatin), vascular remodeling (chemerin) and many more [36].

**Fig. 1:**
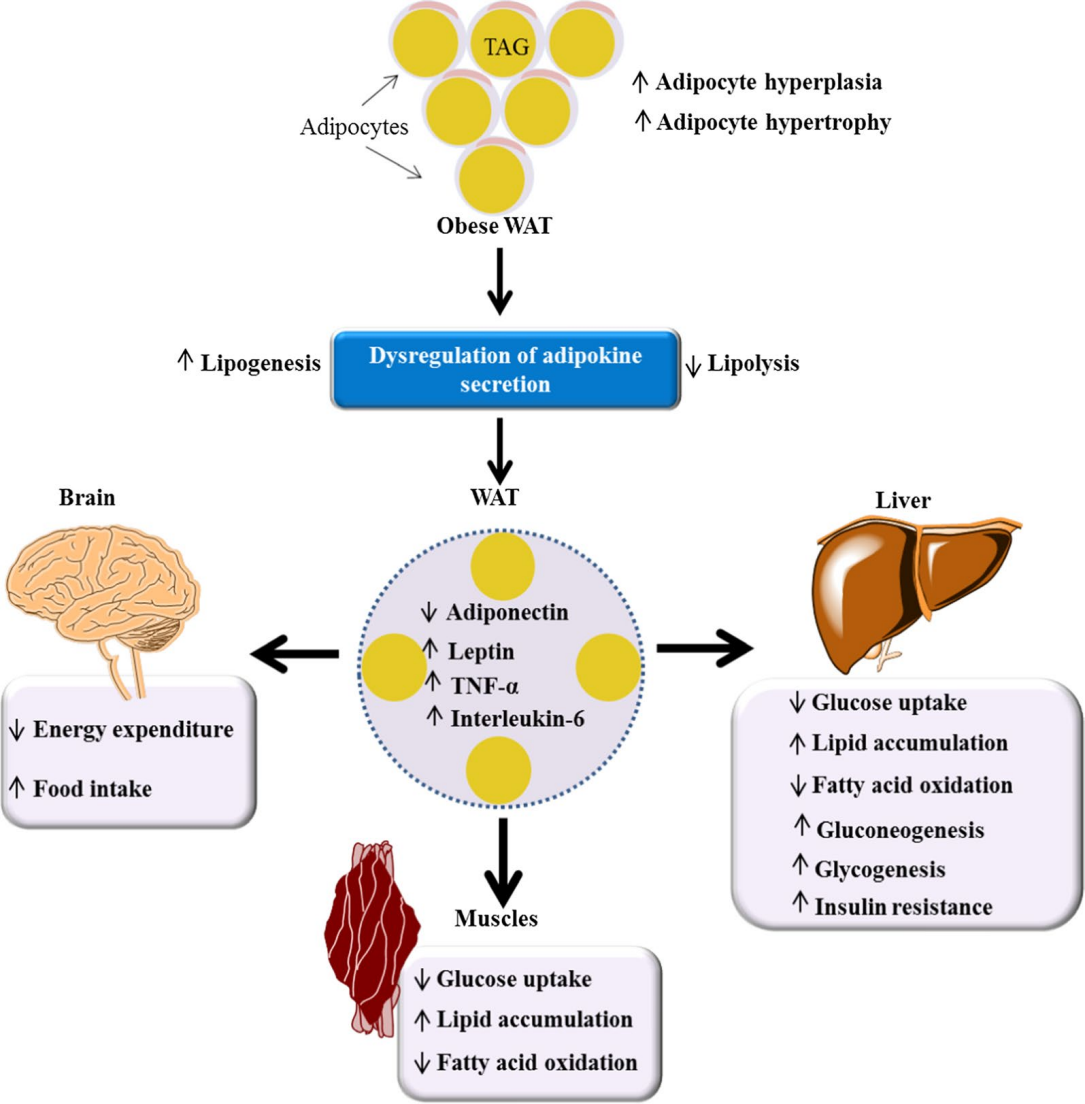
Impact of obesity on the body’s homeostasis. Obesity causes WAT dysfunction and alters adipokine expression. Changes in adipokine expression in the WAT, affect the activity of other organs (brain, liver and muscles)

The WAT is central in the development of obesity and its associated diseases. As a consequence of chronic intake of excess energy, the WAT expands by increasing the number of pre-adipocytes (hyperplasia) and the size of adipocytes (hypertrophy) [36]. Obesity alters the WAT functions and homeostasis, whereby the changes in adipokine expression affects the activity of other major organs such as brain, liver, kidney, heart and muscles (Fig. 1). Alteration of the physiological activities in these organs is accompanied by development of secondary diseases [4, 5], for instance chronic ectopic fat in the liver could lead to development of hepatic steatosis [49]. These adipokines are used clinically to assess the presence or absence of obesity-induced diseases, as well as targets for therapeutic intervention [50, 51]. Some of them have been explored in preclinical studies as targets for drug therapy and to assess progression to other diseases [11, 13, 14, 16]. Therefore, strategies that are targeted at the WAT to reduce its size, destroy the hypertrophic adipocytes, transform WAT to BAT or inhibit adipogenesis could be ideal not only for obesity treatment but its associated comorbidities as well.

## Targeted nanotherapy for treatment of obesity

Pharmacotherapy still remains an area of ongoing research in search for a magic anti-obesity drug that will have significant and sustainable weight loss effects. Since the introduction of thyroid hormone as the first anti-obesity drug in 1893, most of the drugs that followed have been withdrawn due to their adverse side effects in obese patients [1, 2, 5, 29, 52]. Thus, extreme caution is mandatory before considering any drugs for the treatment of obesity. In addition to the drug’s efficacy, its safety profile is equally as important. In recent years, experimental targeted therapy has been reported to promote substantial weight loss with reduced side effects in animal models of obesity [11, 53]. These anti-obesity effects were enhanced by using nanotechnology-based delivery systems [13–19]. Three nano-based strategies that focused on the WAT and its vasculature as a target for obesity therapeutic intervention are reviewed here.

### Inhibition of angiogenesis in WAT reverses obesity

It is well known that neovascularization (angiogenesis, formation of new blood vessels) and adipogenesis (formation of new fat cells) are temporally and spatially coupled processes [6, 7, 54]. Due to its plasticity and constant remodeling, the WAT is highly vascularized and depends on an extensive blood supply systems (vasculature) for sustained growth and expansion [54]. During development of obesity, the tissue expands by both hyperplasia of preadipocytes and hypertrophy of adipocytes [54, 55]. As the tissue continues to grow, it triggers the release of pro-angiogenic factors that promote extension of the pre-existing blood vessels towards the diseased cells in order to supply the tissue with nutrients and oxygen [6, 54]. This occurs through a process called angiogenesis. Regulating WAT growth by targeting its vasculature with anti-angiogenic inhibitors is therefore a feasible strategy to treat obesity [6, 54, 55]. Pro-angiogenic factors that were identified in the WAT as targets for the treatment of obesity and therapeutic intervention include vascular endothelial cell growth factor (VEGF), fibroblast growth factor, angiopoietin, leptin, platelet derived growth factor, matrix metalloproteinases, cathepsin D, hepatocyte growth factor [7, 54], and PHB [11, 54]. Unlike in the WAT where angiogenesis promotes energy storage, angiogenesis is essential for BAT hyperplasia for the rapid cell division of brown fat precursor cells and endothelial cells for energy expenditure [6, 7]. Therefore, it is crucial that anti-angiogenesis treatment be targeted in order to avoid bystander effects, because angiogenic components involved in adipogenesis are also critical to many other biological processes [6, 7].

**Fig. 2:**
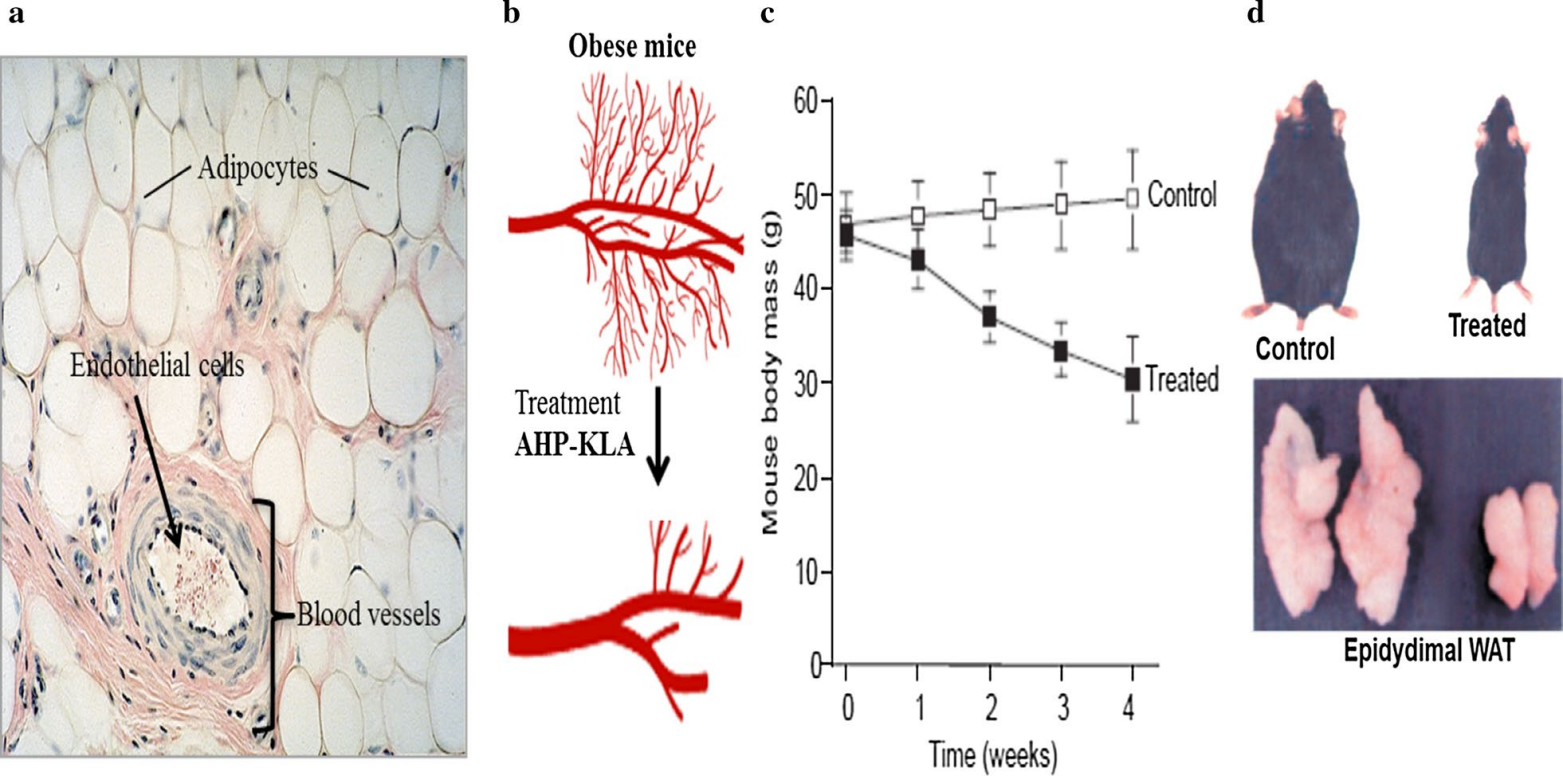
Reversal of obesity using PHB-targeted pro-apoptotic agent. The WAT is highly vascularized, during development of obesity it expresses pro-angiogenic markers that can serve as a target for treatment of obesity (**a**). Targeting the WAT vasculature of obese mice with AHP-KLA reduced vascularization associated with obesity (**b**). This was accompanied by reductions in body weights (**c**) and WAT mass (**d**). Reproduced with permission [11]. Copyright 2014, Nature journals

PHB has shown potential as a WAT vascular marker [11]. Although the protein is expressed in various cellular compartments [56, 57], it is highly expressed as a cell surface receptor in the vasculature of the WAT in diet-induced animal models of obesity and humans [11, 13–15]. As such, PHB can be used for targeted delivery of cytotoxic agents to vascular endothelial cells inhibiting angiogenesis in the WATs and reverse obesity (Fig. 2) [11, 57]. Molecules that bind to PHB with high specificity have been identified through phage display [11, 57], among them the CKGGRAKDC peptide has been used in various obesity models [11, 13–15]. This peptide will be referred to as adipose homing peptide (AHP) [15]. Systemic injection of AHP conjugated to a pro-apoptotic _D_(KLAKLAK)_2_ peptide (AHP-KLA, adipotide) in diet-induced obese mice [11] and monkeys [53] resulted in significant body weight loss and reversal of obesity. The chimeric peptide (AHP-KLA) was able to bind to PHB receptor overexpressed by the endothelial cells in the WAT vasculature of obese subjects (Fig. 2a). AHP-KLA was internalized by receptor mediated endocytosis, and once inside the cells the KLA peptide disrupted the mitochondrial function and caused cytochrome C release. Cytochrome C triggered caspase-3 activation that resulted in cell death through the intrinsic apoptotic pathway. Consequently, the treatment normalized the WAT vasculature (Fig. 2b), the metabolic activity of the animals and reduced body weight (Fig. 2c) [11, 53]. The body weight loss was attributed to the reduced WAT mass (Fig. 2d) in both the visceral and subcutaneous fat depots [11]. Therefore, targeting the WAT vasculature could potentially be used to treat obesity and its medical consequences. However, chimeric peptides are prone to degradation and higher dosage will be required to attain efficacy at the target tissue or organ [11]. Moreover, long term systemic circulation may also cause unwanted immune responses and produce antibodies against the chimeric peptide, thus causing reduction of the desired efficacy.

To overcome the protein degradation, immune response and dosage hurdles, nanotechnology-based strategies were developed. Nanotechnology is defined as the development of materials with a size of ≤ 100 nm [57, 58]. Various types of nanoparticles (NPs) have been developed for a number of applications in environmental, energy, water and medical sectors. The choice of nanomaterial is dependent on the intended application. At a nanometer scale, the NPs portray unique physico-chemical properties compared to their bulk counterpart [58–60]. These properties contribute to the novel applications of the nanomaterials in different sectors. In biomedical research, the NP size can be exploited as multimodal systems which allow attachment of multiple molecules on their surface due to their larger surface area [59, 60]. Various NPs for biomedical applications range from lipid based, polymeric and metallic nanomaterials [61]. The specificity and drug efficacy can be enhanced by attaching targeting molecules (antibodies, aptamers, peptides) that recognise disease-specific biomarkers. Therapeutic agents (drugs), and imaging agents to monitor disease response to the treatment can also be attached in the same NPs [62]. Targeted nanotherapy has demonstrated improved therapeutic index, ability to discriminate between diseased and healthy tissues, and a potential to maximize the safety and efficacy of the drugs [13, 14, 62]. Independent studies have reported the feasibility of PHB-targeted NPs to target [13–15, 63] and inhibit angiogenesis in the WAT of diet-induced animal models of obesity [13, 14].

#### Targeted delivery and biodistribution of PHB-NPs

**Fig. 3:**
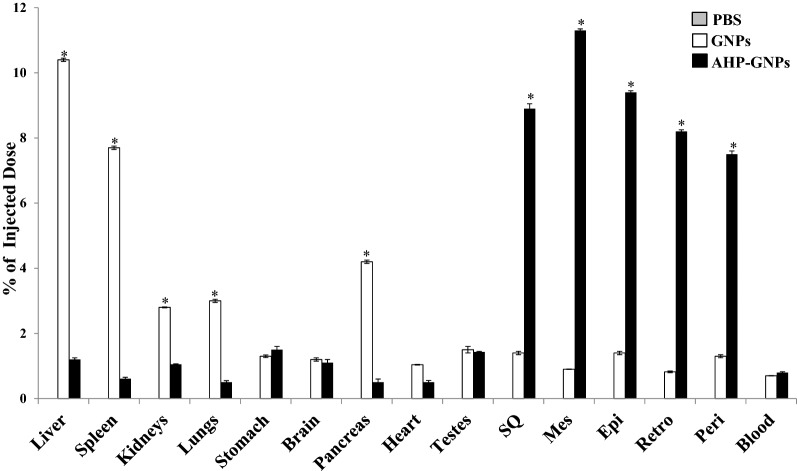
Biodistribution of AuNPs in tissues and organs collected from diet-induced obese Wistar rats. AHP-AuNPs accumulated in PHB expressing tissues (WATs) 24 h post injection, while the untargeted AuNPs mostly accumulated in the RES organs. Reproduced with permission [13]. Copyright 2015, Springer. *PBS* phosphate buffered saline, *GNP* gold nanoparticles, *AHP* adipose homing domain, *SQ* subcutaneous, *Mes* mesenteric, *Epi* epididymal, *Retro* retroperitoneal, *Peri* perirenal, *WATs* white adipose tissues

**Fig. 4:**
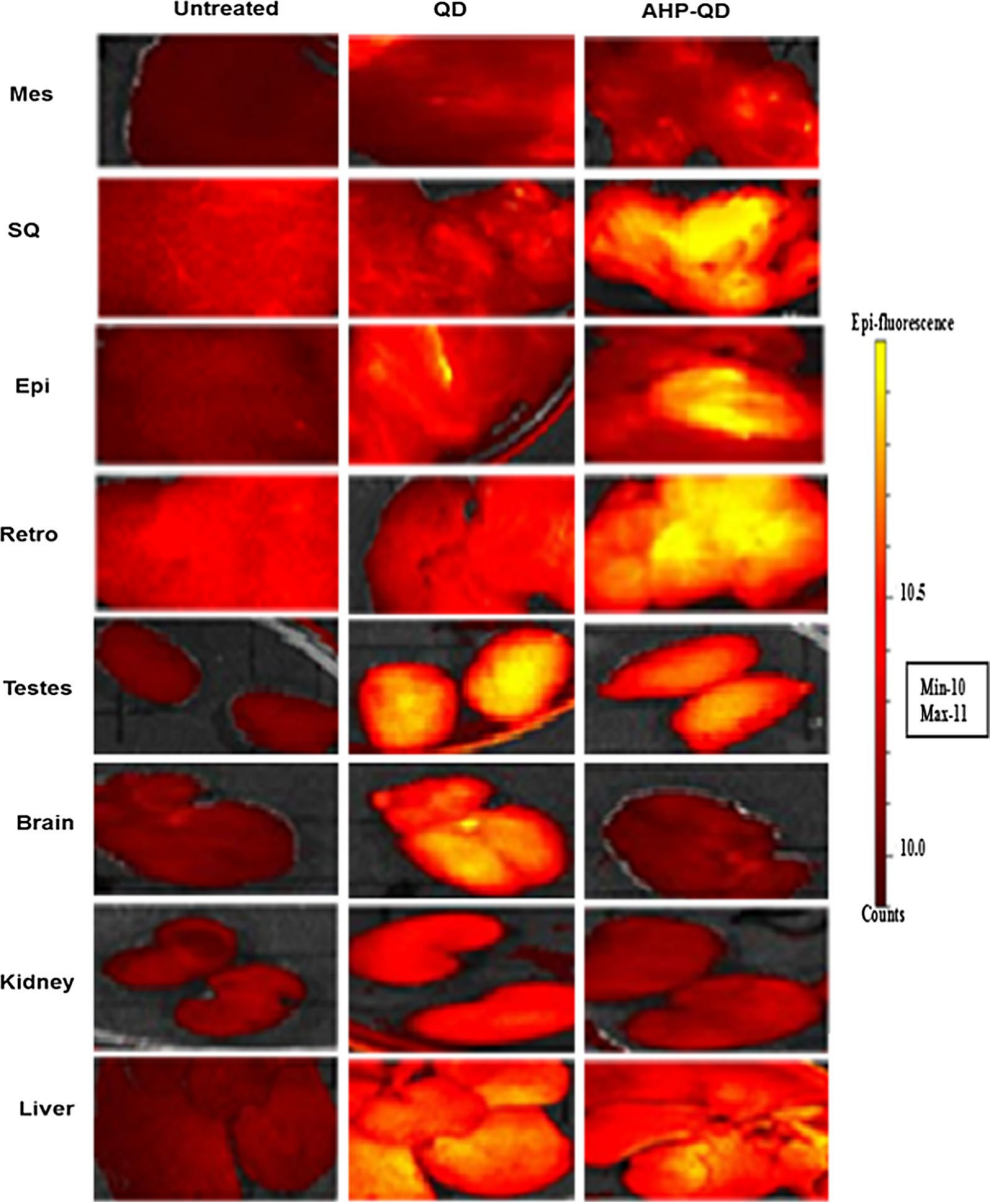
Biodistribution of QDs in diet-induced obese Wistar rats tissues and organs. AHP-QDs accumulated in PHB expressing tissues (WATs) 24 h post injection, while the untargeted QDs mostly accumulated in the RES organs. Reproduced with permission [63]. Copyright 2018, Dove Medical Press. *QD* quantum dot, *AHP* adipose homing domain, *SQ* subcutaneous, *Mes* mesenteric, *Epi* epididymal, *Retro* retroperitoneal, *Peri* perirenal

The specificity and biodistribution of gold NPs (AuNPs) [15] and quantum dots (QDs) [63] were demonstrated in a diet-induced obese Wistar rats. The rats were intravenously injected with a single dose of either untargeted (peptide-free AuNPs [15] and QDs [63]) or PHB-targeted NPs (AHP-AuNPs [15] and AHP-QDs [63]), and sacrificed 24 h post-injection. Analysis of tissues by inductively coupled plasma optical emission spectroscopy (ICP-OES) (Fig. 3**)** showed the selectivity of the AHP-AuNPs for PHB-expressing tissues (WATs) while the peptide-free AuNPs accumulated mostly in the reticuloendothelial system (RES) organs such as liver, lungs, spleen, kidney [15]. Similar observations were made with the QDs (Fig. 4), where the fluorescent signal of AHP-QDs was detected in the WATs, and that of the free QDs was observed in the RES organs using Xenogen imaging system [63].

The two studies substantiated that metallic NPs can be delivered into the target tissues, serving as effective drug delivery [15], as well as imaging systems [63] without compromising the functions of their cargoes. These findings were further validated on PHB-expressing cells, the breast (MCF-7) and colon (Caco-2) cancer cell lines, which were reported to express PHB as a cytosolic and extracellular receptor, respectively [15, 62]. These cells demonstrated the sensitivity and specificity of the PHB-targeted AuNPs containing KLA pro-apoptotic molecules (AHP-AuNP-KLA) as a treatment, whereby the targeted nanotherapy induced a significant anti-proliferative activity on the cells that express the receptor for AHP on the cell surface (Caco-2 cells). The therapeutic activity of the KLA peptide was retained and enhanced following conjugation to AuNPs through receptor mediated targeting, and demonstrated differential uptake by Caco-2 cells (cells that express PHB on the cell surface). Thus, targeted therapy could be a plausible strategy for treatment of chronic diseases including obesity [63].

#### Anti-angiogenic effects of PHB-targeted nanotherapy

**Fig. 5:**
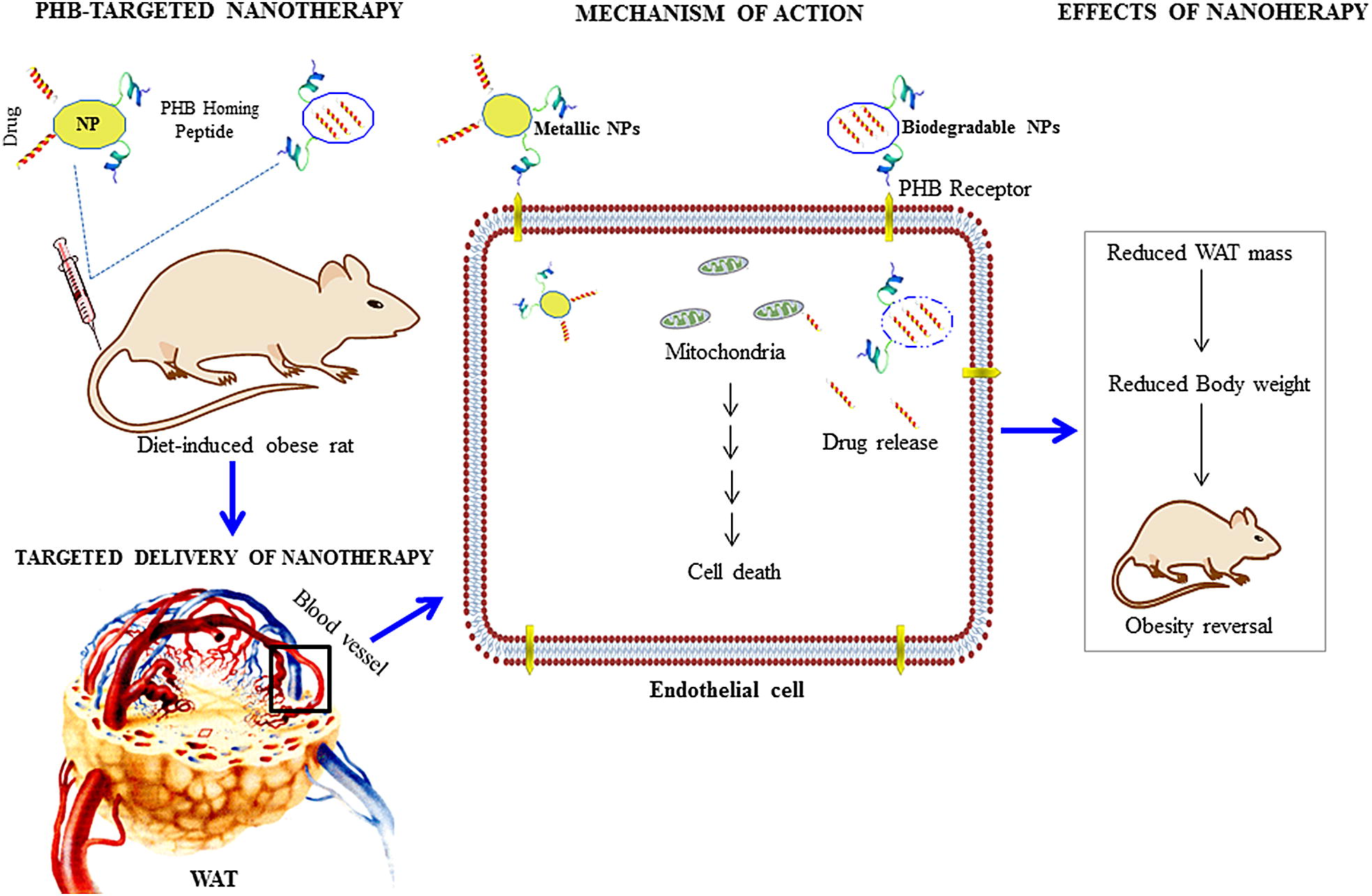
Mechanism of PHB-targeted nanotherapy for reversal of obesity in diet-induced obese rats. The targeted NPs will bind to the PHB receptor on the cell surface. Once the nanomaterials are internalized, the therapeutic peptide will trigger cytochrome C release from the mitochondria, followed by caspase activation then cell death through apoptosis. *NP(s)* nanoparticle(s), *PHB* prohibitin, *WAT* white adipose tissue

Angiogenesis plays a crucial role in the pathogenesis and progression of obesity, hence, strategies that can inhibit angiogenesis in the WATs could potentially be able to reverse obesity. Targeting fat depots using angiogenesis inhibitors (e.g. TNP-470, angiostatin, and endostatin) reduces body weight [6, 11, 54, 55], providing validation that anti-angiogenic strategies may be a useful anti-obesity therapeutic approach. Preclinical animal studies demonstrated anti-obesity effects of AHP-KLA biconjugate in obese mice [11] and monkeys [53], these effects were improved by using nanotechnology-based delivery systems [13–15]. The PHB-vascular targeted nanosysems had reproducible results using various types of nanomaterials such as AuNPs, QDs, liposomes, and polymeric NPs [13, 15, 63]. The mechanism of action of either metallic or biodegradable NPs in obese subjects is summarized in Fig. 5. After intravenous injections, the NPs localize to the endothelial cells by binding to the PHB receptor in the WAT vasculature. Once inside the cells, the KLA peptides on the surface of the metallic NPs are free to interact with cellular organelles while the ones encapsulated in the biodegradable NPs will rely on the cellular environment to trigger its release. This is followed by induction of apoptosis in the endothelial cells by the KLA peptides which then results in reduced WAT mass and total bodyweight. Disrupting the blood supply to the WAT starves the adipocytes, forcing them to metabolize the excess energy possibly through lipolysis. Another assumption could be through adipocyte cell death since not enough oxygen can reach these cells [13, 14].

The nanocarriers significantly enhanced the potency of the therapeutic peptide (KLA), increased drug uptake and accumulation in the target site by either enhanced permeability and retention (EPR) effect [13] or receptor-mediated targeting [13, 14]. This dual-targeting mechanism was investigated by comparing the activity of untargeted NPs (KLA-liposomes) against that of PHB-targeted nanotherapy (AHP-KLA liposomes) on obese mice. PHB-targeted nanotherapy was developed by encapsulating KLA peptides in liposomes, and attachment of AHP on the NP surface. KLA-liposomes used passive targeting based on the tissue’s EPR effect [13], whereas AHP-KLA liposomes bound to the PHB receptor and entered the cells via active targeting. Both systems managed to target and destroy WAT angiogenic blood vessels, resulting in reduced body weight. Postmortem analysis revealed that weight loss was due to decreased mass in the subcutaneous (inguinal) and visceral (epididymal) fat depots. The nanosystems, in addition to their anti-obesity effects, showed potential for prevention of obesity-induced metabolic diseases. The animal ‘s metabolic functions were normalized by reduction of the ectopic fat accumulation in the liver and muscles [13, 14]. Moreover, the nanosystems were also biocompatible and showed no indication of toxicity after 4 weeks of treatment [14]. When compared to AHP-KLA (3 mg/kg body weight), at a threefold lower dose of untargeted and PHB-targeted KLA liposomes (1 mg/kg body weight) the anti-obesity effects were significantly enhanced. Another advantage of using the nanosystems was that more drugs can be incorporated in one NP, while the chimeric peptides deliver one KLA peptide per one AHP molecule. Furthermore, drug release from the NPs can be controlled and have longer circulation time and prolonged release [14].

### Transformation of WAT into BAT increases thermogenesis

The function of BAT as an energy dissipating organ is well established, together with its ability to produce heat energy and increase thermogenesis (energy expenditure) [3, 35, 39, 42, 43, 64]. The recent appearance of beige or brown-like adipocytes in WAT depots of adult humans has sparked interest to use thermogenic activity for obesity treatment [38, 45]. This has led to exploration of WAT browning as a target for obesity treatment [39, 42, 43, 64]. Hence, strategies that can stimulate the uncoupled respiration rate and thermogenesis through reactivation of BAT or transformation of WAT to BAT could provide alternative approaches for treatment of obesity and its comorbidities [23, 38, 40, 44, 64].

The brown-like (beige or brite) adipocytes detected in human WAT behave and function like the brown adipocytes [38, 42, 43]. Their UCP-1 levels and mitochondrial content are comparable to those of brown adipocytes; and through uncoupled mitochondrial respiration, the beige adipocytes can also burn fat and generate heat, increase energy expenditure and reduce body weight [23, 38, 44, 64]. WAT browning can be induced through two main mechanisms, either by control of environmental temperature (cold exposure) or sympathetic nerve innervation (β-adrenergic signaling) [23, 64]. Cold exposure-related browning occurs as an adaptive response through non-shivering thermogenesis. After prolonged exposure to cold temperatures (4–16 °C) in experimental settings, the body attempts to sustain the core temperature by increasing its metabolic activity [46–48]. At this state, transcription factors (peroxisome proliferator-activated receptor (PPAR) γ and α, PPARγ-coactivator-1α and PR domain-containing protein 16) that are involved in uncoupled respiration become activated, which then trigger WAT browning by increasing the UCP-1 expression [38, 46, 47]. Natural cold exposure-related browning is mediated by sympathetic nervous system through norepinephrine signaling. Norepinephrine, secreted by the postganglionic sympathetic neurons binds to β-adrenergic receptor and initiate cold-induced thermogenesis [38, 41, 47]. β-adrenoceptor agonists have been used in browning of both subcutaneous and visceral WAT depots. However, these receptors are also found in the gastrointestinal tract, prostate and bladder [23, 65] and its variants in the heart and kidneys [23]. And more specific agonists will be required to avoid off target browning.

#### WAT Browning agents

Various agents capable of inducing WAT browning have been reported, and only the three highlighted in Table 1 will be discussed in this review. They are currently used as treatment for diseases other than obesity, and their browning activity is now explored as a possible anti-obesity treatment.

**Table 1:** The actions and limitations of WAT browning agents

Browning agents	Actions	Limitations	Current application	Refs
Dibenzazepine	γ-Secretase inhibitor Inhibit Notch signaling Act in the liver to improve systemic metabolism	Solubility Non-specificity	Anti-depressant	[17]
Resveratrol	Sirtuin activator	Solubility Stability Metabolic transformation Less bioavailability High metabolism in humans	CVDs	[23, 66]
Rosiglitazone	PPAR gamma activator	Hydrophobic Solubility	T2D	[12]

#### Dibenzazepine induce WAT browning by inhibiting Notch signaling pathway

Dibenzazepine (DBZ) is an iminostilbene. Drugs in this category are used as anti-depressants, mainly for treatment of schizophrenia [17]. It exerts its actions by blocking the activity of γ-secretase, leading to inhibition of the Notch signaling pathway. Notch signaling is very crucial in the development of embryos, tumorigenesis, vascular remodeling, and determines the fate of stem cells. Dysregulation in this process has been implicated in the development of inflammatory diseases [67]. Notch signaling activity is extremely high in pathological WATs associated with metabolic syndrome, especially in obesity, diabetes and CVDs [68]. Therefore, suppression of Notch signaling has beneficial health effects against a wide range of metabolic and inflammatory diseases [17, 67].

The role of Notch signaling in obesity is still elusive [64], however some studies have shown that inhibition of this process in the WAT by a γ-secretase inhibitor (DBZ), reverses obesity and improves metabolic functions [17, 64]. DBZ induced anti-obesity effects by transforming the WAT to BAT [64]. However, the use of free DBZ can be accompanied by side effects, as failure to deliver the drug at the target location will cause biogenesis of brown adipocytes and inhibition of Notch signaling in all cells they come into contact with [17]. These undesirable effects might induce development of secondary diseases [64]. Local delivery of DBZ to target tissues was achieved by using poly(lactide-co-glycolide) (PLGA) NPs as drug delivery agents. Encapsulation of DBZ in PLGA NPs improved its targeting and led to localization of the drugs on target tissues. Three days post intraperitoneal injection of the DBZ-loaded NPs directly into the subcutaneous WAT depot, the untargeted NPs were retained at the site of injection. Although the NPs managed to spread throughout the local tissue, no NPs were detected in other tissues including the RES organs. Using this carrier system confined the DBZ browning effect only in the target tissue, enhanced energy expenditure and prevented weight gain in the diet-induced obese mice. The metabolic activities of the animals were also normalized as evidenced by reduced blood glucose, cholesterol, insulin, TAGs and free fatty acids [17]. The improvement in insulin sensitivity and glucose tolerance in the animals, suggest that the browning effect through nanosystems can also be used for treatment of metabolic syndrome [17].

#### Resveratrol-induced WAT browning

Resveratrol (3,5,4′-trihydroxy-trans-stilbene, Res) is a natural polyphenolic compound mostly found in grape skins and red wine, although it can also be produced by other plants such as berries and peanuts [38, 68]. It is a phytoalexin produced by plants in response to attacks by either bacteria or fungi as a defense mechanism. Its health benefits as an antioxidant, include protection against CVDs, cancer, diabetes, and Alzheimer’s disease [69]. It is also used as one of the main components in the Kojo-kon medicine, a traditional medicine used in the treatment of heart and liver diseases. It has earned its market name of the “French paradox” because it has shown low incidences of heart diseases among French people who indulge in high fat diets [68, 69].

Of interest, Res is one of the potential anti-obesity drugs currently in phase II clinical trials [23, 70]. The preclinical anti-obesity effects of Res have been reported, which are mainly attributed to its ability to regulate adipocyte differentiation, lipolysis, mitochondrial biogenesis and fat acid oxidation [71, 72]. Other studies also demonstrated that Res administration can enhance expression of BAT genes such as UCP-1 and CD137, suggesting that it is responsible for transformation of WAT into BAT or beige-like adipocytes, leading to increased energy expenditure and reduced body weight [44, 71, 73]. An independent study demonstrated the synergistic effect of Res as a food supplement. Incorporating stilbene synthase (Res biosynthesis gene) in Dongjin rice through recombinant DNA technology improved the endogenous anti-obesity effect of the rice [74]. However, Res has poor solubility in water (< 1 mg/mL), is prone to undergoing metabolic transformation (methylation or glucoronidation) in the liver, and has high metabolism in humans which reduce its stability, bioavailability and activity [73–77].

Various types of nanosystems can used as delivery system to increase Res’s bioavailability and stability. Encapsulation of Res in lipid nanocarriers and liposomal delivery systems was able to increase its solubility, distribution and bioavailability in vitro studies. Res is sensitive to light and high temperatures. The lipid nanocarriers have been shown to mask the drug (Res) from degradation and prolonged its release. The browning activity of Res-loaded NPs on 3T3-L1 adipocytes was retained as evidenced by the increase in expression of beige (CD137) and brown adipocytes markers (UCP1, PPARγ co-activator 1α), coupled with reduced expression of white adipocytes markers (insulin-like growth factor-binding protein 3) [66, 76]. Similar effects were achieved by Res-loaded PLGA NPs for treatment of non-alcoholic fatty liver disease. Both the empty vehicle and Res-loaded PLGA NPs were biocompatible and showed no toxicity towards the HepG2 cells during the 24 h test period. Interestingly, the NPs accumulated in an in vitro model of oleic acid-induced hepatic steatotic (HepG2) cells, inhibited lipid accumulation and lipogenesis, while enhancing lipolysis [78]. An independent study also showed the potential of self-nano-emulsifying drug delivery system to protect and enhance the bioavailabilty and pharmacokinetics of Res oral formulation [79]. For clinical applications, Res as a multifunctional compound presents a major challenge due to lack of specificity, and its multiple actions on adipocytes [73], hepatocytes [78], cardiomyocytes [69], etc. In order to improve its specificity, the attachment of targeting molecules can be used to restrict its functions to target cells. This was further demonstrated by attaching transferrin antibodies on solid lipid NPs loaded with Res. The targeted NPs selectively bound and accumulated on the endothelial cells, these cells express transferrin receptors and used as a human blood brain barrier model for Alzheimer’s disease [77].

#### Rosiglitazone promotes WAT browning by activating PPARγ

Thiazolidinediones (TZDs) such as troglitazone, pioglitazone, and rosiglitazone (Rosi) are potent anti-diabetic agents. TZDs bind to the nuclear peroxisome proliferator-activated receptor (PPAR)-γ, subsequently activating genes that encode proteins involved in the metabolism of glucose and lipids. This leads to an increase in glucose uptake in skeletal muscle and adipose tissue, reduction in hepatic glucose output, and finally, an increase in free fatty acid uptake. Besides their anti-diabetic potency, these TZDs have anti-inflammatory effects on vascular cells. Rosi is TZD, currently used as an anti-diabetic drug alone (Avandia®) or in combination with either metformin (Avandamet®) or glimepiride (Avandaryl®). Health effects of Rosi include anti-inflammatory effects, enhanced insulin sensitivity [80, 81], and anti-Alzheimer activity [82]. PPARγ is a nuclear transcription factor expressed in the WATs, pancreatic β cells, vascular endothelium, and macrophages [81, 83]. In the WAT, it is involved in adipocyte differentiation, fatty acid uptake and storage, and glucose uptake. Rosi has a number of side effects that are observed in at least 5% of patients, which include upper respiratory tract infection, headache, edema, weight gain, risk of heart attack and death [81].

Despite its contradictory effect of increasing body weight in diabetic patients [83, 84] Rosi was also reported to have anti-obesity effects resulting from its ability to induce WAT browning [12, 16, 85], which in turn activate angiogenesis, increase mitochondrial biogenesis and cause weight loss in rodents. As such, Rosi showed potential as an anti-obesity drug. Rosi blocks the actions of tumor necrosis factor (TNF)-α in the WATs, as a result increases adipocyte differentiation (adipogenesis) and reduce release of free fatty acids from adipocytes [81, 83]. Rosi is administered orally, and while it is easily absorbed, the majority of the drug tend to bind to blood proteins, thus reducing its efficacy. Since most of the drug is metabolized by the cytochrome P-450 2C8 isozyme, and excreted in the urine [81], higher dosages of the drug is required to reach the target tissues. Interestingly, nanocarriers can control the rate at which the drugs are released by making them sensitive to certain physiological parameters such as pH, enzymes, and temperature, in order to trigger drug release once they are inside the cells. This was demonstrated by encapsulating Rosi on polymeric [12] and glucose-responsive dextran [16] NPs as discussed below.

#### Rosi-NPs stimulate angiogenesis and enhance WAT browning

Inhibition of angiogenesis in the WATs enhances lipolysis and reduces body weight [11, 13, 14, 54], while the opposite occurs when using the strategies that reverse obesity by stimulating energy expenditure [12]. Active angiogenesis is crucial for highly thermogenic cells, which in the WATs can lead to tissue browning and body weight loss. Rosi has dual functions in the reversal of obesity: stimulation of angiogenesis and transformation of WATs. These effects are confirmed by reversal of obesity through vascular targeted Rosi NPs. Polymeric NPs (made up of PLGA-*b*-PEG copolymer) were used to deliver Rosi in order to reduce the bystander effects and enhance its efficacy. The Rosi-encapsulated NPs were localized in the WAT vasculature and increased specificity by targeting PHB or integrin_α_v receptors using AHP and iRGD (CRGDK/RGPD/EC) targeting peptides, respectively. The targeting peptides recognize angiogenic receptors in the WATs. The iRGD peptide is a dual-targeting peptide that is further cleaved into the CRGDK fragment after binding to integrin receptors. CRGDK peptide fragment then bind to neuropilin-1 in the local tissue and enable delivery, entry and uptake of the NPs with its cargo. Mice treated with the vascular targeted-Rosi NPs showed significant weight loss compared to the ones treated with the free Rosi and untargeted NPs. Weight loss was attributed to the enhanced WAT browning and angiogenesis, as confirmed by increased levels of BAT (UCP-1, transcriptional coactivator Cidea, type 2 deiodinase), and endothelial cell angiogenic (CD31, Isolectin B4) markers. Treatment with Rosi-NPs led to phenotypic changes in the inguinal and epididymal WATs, which includes color changes (reddish vasculature and brown adipocytes), reduced adipocyte size, and condensed cellular contents. The targeted Rosi NPs reduced the levels of cholesterol, TAGs, and insulin in the blood, effects which were not detected in mice treated with free drug (Rosi) or untargeted NPs [12].

#### Transcutaneous delivery and WAT browning effects of a microneedle Rosi-NPs patch

A non-invasive microneedle patch was used as a transdermal delivery agent for Rosi-NPs to the subcutaneous WAT. The patch was composed of a drug (Rosi), glucose oxidase and catalase which were encapsulated into biodegradable acid-sensitive dextran NPs. Glucose oxidase was used to convert host glucose into gluconic acid and hydrogen peroxide (H_2_O_2_), thus creating an acidic environment for drug (Rosi) release. Catalase was used to remove the byproduct (H_2_O_2_) of the glucose oxidase reaction. The NPs were then embedded in a polymeric microneedle array patch, made of hyaluronic acid (HA) to enable entry through the skin [16]. HA can pass through the skin’s protective layer, the stratum corneum, which protects internal tissues against desiccation, infection, xenobiotic chemicals, and mechanical stress. In so doing, HA can deliver drugs into the subcutaneous layer [86] and bloodstream [87]. It is used topically for transdermal drug delivery of poorly bioavailable therapeutic agents [87, 88], and has been successfully used in transporting various molecules including growth factors, interferon and anticancer drugs [87, 89].

**Fig. 6:**
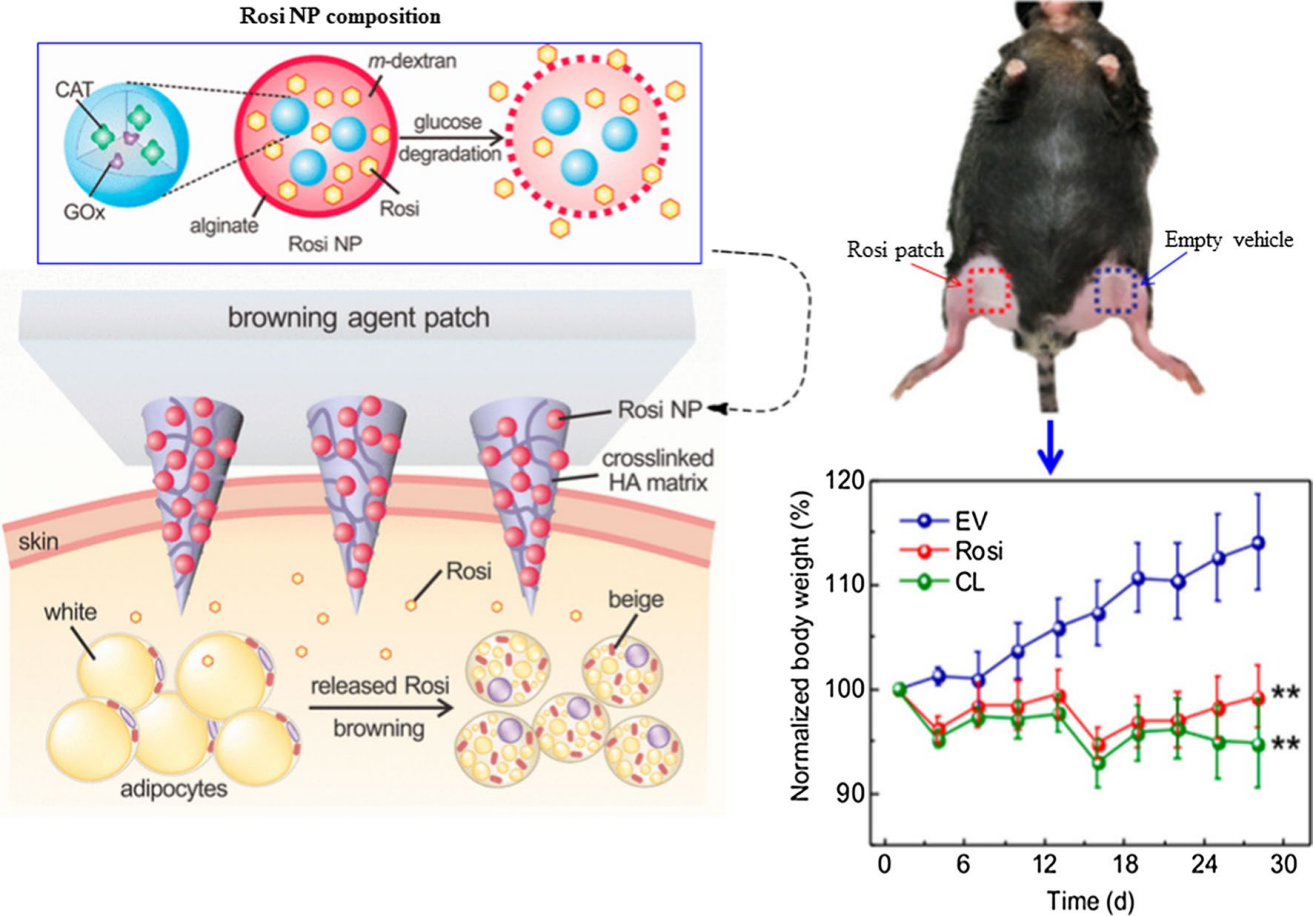
Transformation of subcutaneous WAT into BAT using transcutaneous microneedle Rosi-NP patches. Rosi-NP were prepared by encapsulating Rosi, glucose oxidase (GOx) and catalase (CAT) in pH responsive dextran NPs coated with alginate. The glucose-responsive dextran NPs (Rosi NP) reduced animal bodyweight through browning of WAT by encapsulated Rosi, these effects were comparable to a potent thermogenic activator (CL, CL 316,243). The difference was statistical significant compared to rats subjected to empty vehicle patches (EV). Reproduced with permission [16]. Copyright 2017, American Chemical Society

The therapeutic potential of the microneedle patches was assessed on diet-induced obese mice. The Rosi-NP patches were placed on one side of the inguinal tissue and an empty patch was placed on the other side of the mice (Fig. 6). The glucose-responsive Rosi NPs locally induced subcutaneous WAT browning in the obese mice, which was affirmed by overexpression of BAT markers (UCP1, Dio2, Elovl3, Cidea, PPARγ co-activator 1α, cytochrome c oxidase subunit (Cox) 7a1, and Cox8b. The localization and transdermal delivery of Rosi-NPs was accompanied by phenotypic changes in the target tissues, metabolic changes and weight loss. The study demonstrated that transcutaneous delivery systems could be as effective and produce a comparable therapeutic effect in vivo as the other two strategies that were described earlier. It also presents a more favorable and desirable delivery strategy as it is non-invasive, painless and does not require trained personnel to administer treatment. In addition to body weight loss through localized browning of the inguinal WAT, the treatment also reduced visceral WAT, reduced interleukin-6 inflammatory marker and enhanced metabolic activities [16].

## Nanotechnology-based photothermal lipolysis

Photothermal therapy (PTT) involves the use of photothermal agent which can be excited by a laser to generate heat energy that in turn destroy and kill malignant cells. PTT-induced cell death occur via two major pathways either by necrosis or apoptosis [90]. During necrosis, the heat disrupts the plasma membrane, causing cell burst and leakage of cellular components. This results in inflammation and damage to the surrounding cells/tissues [90]. Apoptosis on the other hand, occurs in a controlled manner with reduced inflammatory activities, and therefore more desirable for clinical application [91].

**Fig. 7:**
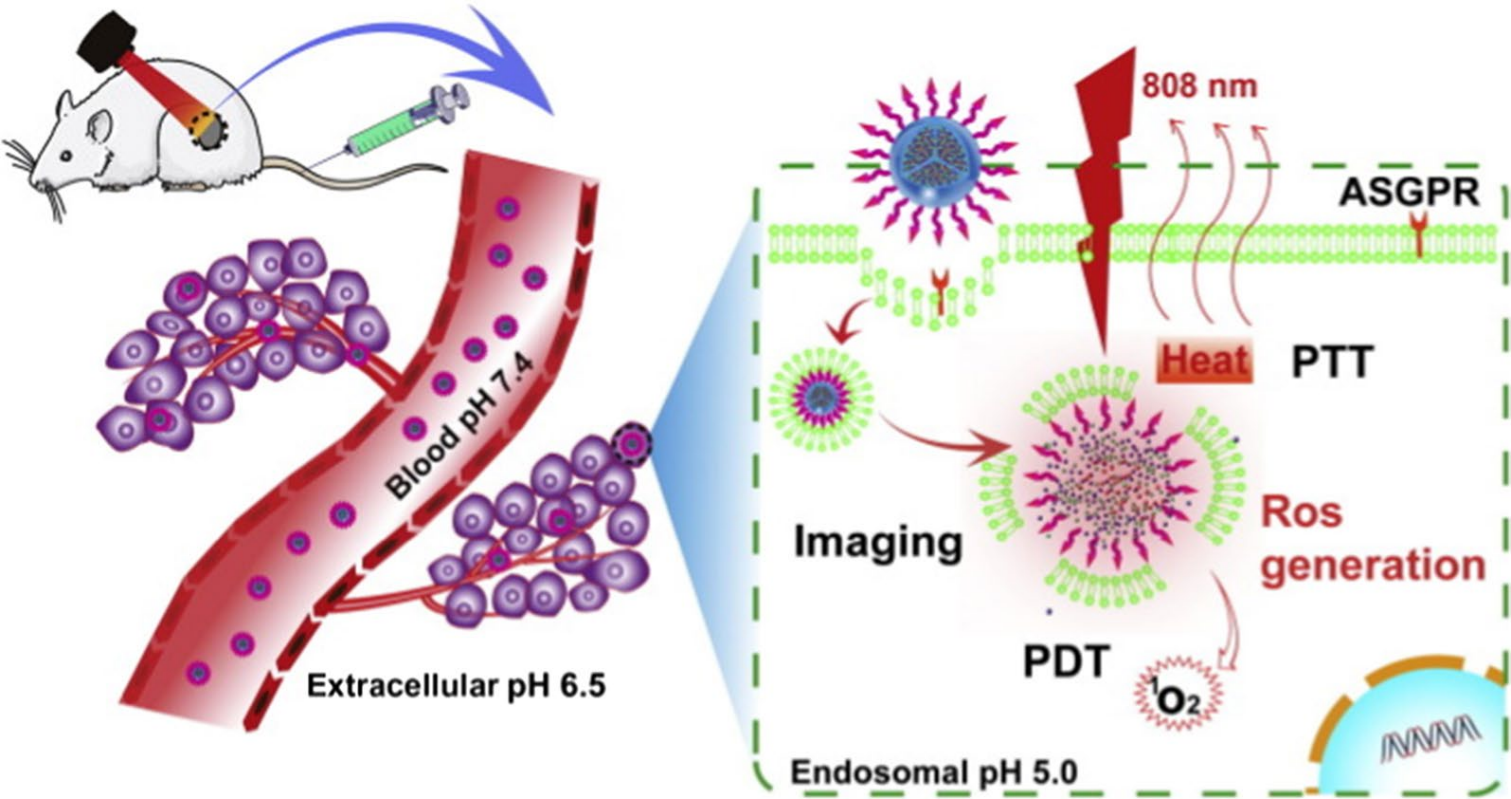
Nano-based PTT for treatment and imaging of diseases. The carrier-free self assembly nanodrug conjugate selectively accumulated in the diseased site after intravenous administration of the nanodrug conjugate, and induced cell death following PTT after laser exposure. Reproduced with permission [95]. Copyright 2018, Elsevier. *PDT* photodynamic therapy, *PTT* photothermal therapy, *ASGPR* Asialoglycoprotein receptor

Metallic NPs are effective photothermal agents as they can convert visible or near infrared (NIR) light into heat after excitation by laser, and destroy target cells [90, 92–94]. Figure 7 presents a nano-based PTT mechanism for imaging and destruction of cancer cells, and an alternative carrier free nanodrug system that can deliver theranostic agents to target cells. This system eliminates the uncertainties surrounding the fate of inorganic materials in living systems, as it uses the self-assembly chemotherapeutic drug as a delivery system. In this particular study, a NIR fluorescent dye (indocyanine green) was loaded in ursolic acid self-assembly nanodrug. The carrier-free nanodrug conjugate through lactobionic acid on its surface, was selectively targeted at cancer cells that express asialoglycoprotein receptor. Indocyanine green is the only FDA-approved photosensitizer for clinical use. Its application is limited by poor solubility and short half-life [95].

Metallic NPs as photosensitizing agents are stable, photostable, biocompatible, and non-toxic. As such, metallic nanomaterials holds a great promise for PTT [90, 92–94]. Their shapes play a crucial role in PTT, and can be used to induce selective cell death [94]. Different NP shapes (nanospheres vs nanorods) absorb and emit light differently, and these parameters can potentially be used to regulate the switch between necrosis and apoptosis [94]. Gold nanosphere (AuNS)-induced PTT is suitable for shallow cancers (e.g. skin cancer) [96], and has demonstrated localized photothermolysis of target cells in vitro and in vivo [91, 97–99]. Although visible light is successful to some extent in destroying diseased or malignant cells that accumulate the AuNSs, the need for radiation to penetrate deep into tissues is desired for the clinical application of PTT. NIR external radiation is capable of achieving this, as it can penetrate soft tissues up to 10 cm at the NIR tissue transmission window of 650–900 nm [100]. By changing the shape and composition of NPs from spherical to rod-shaped, the surface plasmon resonance (SPR) is shifted into the NIR transmission window [96, 101]. Attaching targeting molecules on gold nanorods (AuNRs) enhanced localization into target cells, reduced exocytosis, and toxicity in vivo [91]. The nanosystems could be effective photosensitizers in nano-based PTT for treatment of chronic diseases and provide an alternative platform for translation into clinical use.

The benefits of PTT have been explored for obesity treatment. Obesity results from expansion of WATs due to uncontrollable proliferation of adipocytes in various WAT depots, therefore its treatment requires removal/resection of the diseased cells/tissue. This strategy has worked well for removal of tumor tissues [91]. In obesity, laser-assisted liposuction and laser radiation are among the procedures that have been used to reduce WAT mass by taking out excess fat from subcutaneous and visceral WAT depots. Laser radiation demonstrated selective photothermolysis of WAT in vitro as well as in human subjects. Humans were subjected to a 1210 nm laser directly to their abdomens, followed by collection of biopsies the first 3 days and at 4–7 weeks of laser treatment. The biopsies showed laser induced damage of the tissue. This study demonstrated the potential use of laser-based lipolysis for reduction of fat within the WAT, and for treatment of obesity [102]. However, these procedures are highly invasive, uncomfortable, painful, takes a long time to recover, causes deformities, and lumpy skin due to seromas [18, 19, 102]. These procedures can be improved through the use of nano-based thermal agents to reduce discomfort, and laser-induced tissue damage.

### Photothermal lipolysis of WAT: effect of AuNSs vs AuNRs

Metallic NPs have shown potential for photothermal lipolysis of adipocytes in the WAT of diet-induced obese mice. AuNSs and AuNRs can absorb visible and NIR light and convert it to heat energy by SPR, and destroy target cells. The fat tissues have different thermal relaxation rates to its surrounding environment, and so this allows for rapid and localized heating only in the WAT depots [19, 103]. The WATs that have accumulated the NPs will heat up faster, and dissipate heat slower than water, due to its lower specific heat capacity and thermal conductivity. Heating by this mechanism selectively softens and loosens the WAT, facilitates removal of excess fat with minimal trauma. Only the target tissues, where the NP solution is infused, it will absorb laser energy, therefore, minimizing the potential damage to the surrounding tissues [19]. The heat energy generated from the NPs does not radiate any form of energy which might be toxic to the human body. Hence, metallic NPs might provide a safer and healthier PTT for patients than other sorts of targeted radiation techniques that are used for diseases such as cancer [99].

**Fig. 8:**
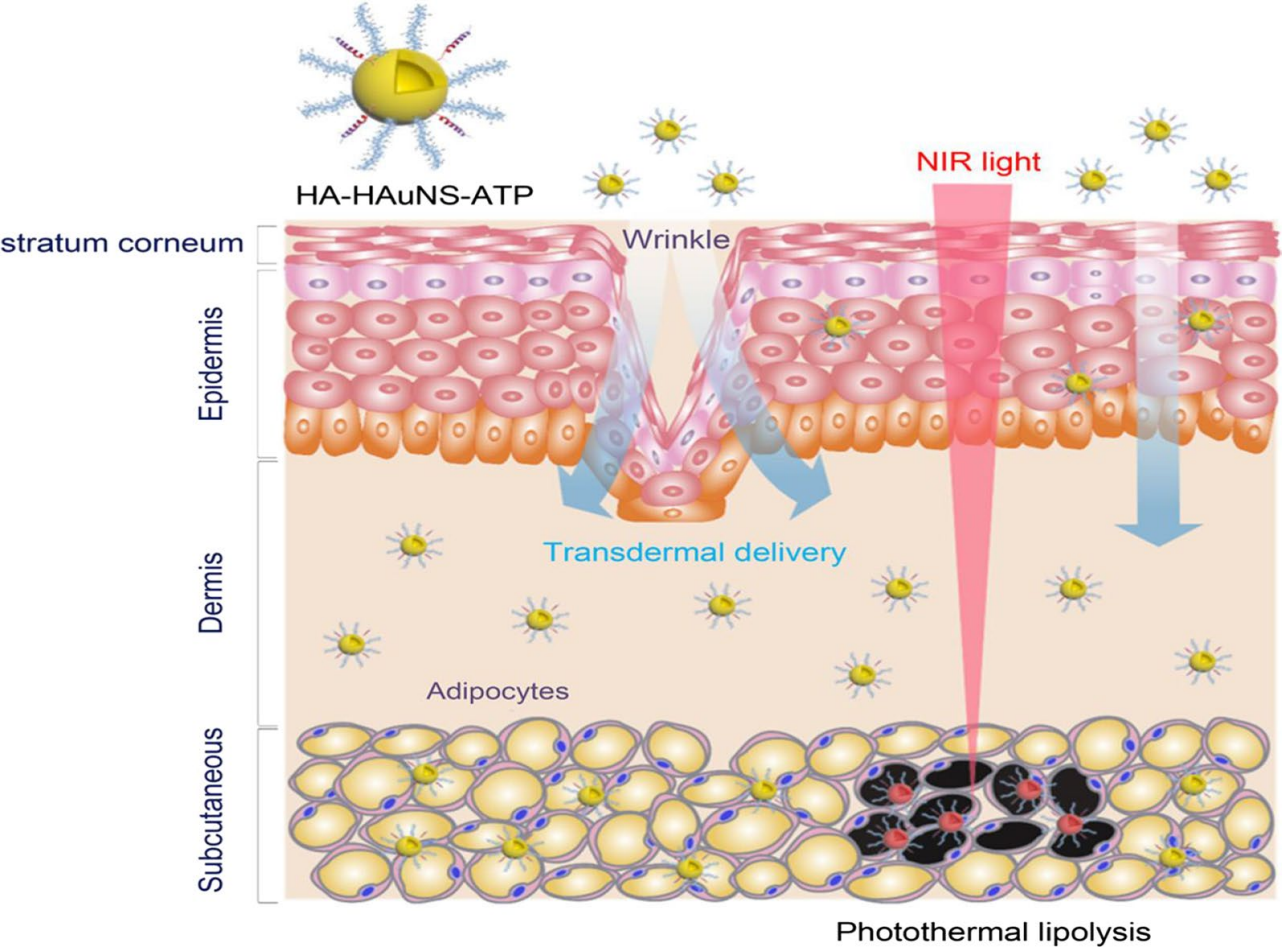
Photothermal lipolysis of adipocytes in the subcutaneous WAT. The AHP-targeted hyaluronate-hollow AuNSs localized in the adipocytes after noninvasive transdermal delivery, and induced photothermal lipolysis in vivo. Reproduced with permission [18]. Copyright 2017, American Chemical Society. *HA-HAuNS-ATP* AHP-targeted hyaluronate-hollow AuNS, *NIR* near infrared

The nano-based systems are especially interesting due to their increased loading capacity, and potential for attaching multiple molecules. Their physico-chemical properties can be altered by attaching various molecules on the NPs. For instance, a dual targeted hollow AuNSs were able to pass through the three layers of the skin non-invasively, and through PTT, was able to induce adipocyte cell death in mice [18]. Hyaluronic acid (HA) attached to the hollow AuNSs allowed transdermal delivery of the NPs to the target tissue. The hyaluronate hollow AuNSs were functionalized with AHP to target PHB once they reach the vasculature of the subcutaneous WAT. The NPs were topically applied on the abdominal region of diet-induced obese mice, transdermal delivery was assessed by photoacoustic imaging. As shown in Fig. 8 [18], the NPs were able to pass through the stratum corneum and dermis, and localize in the subcutaneous tissue. After just an hour, a signal for PHB-targeted NPs was reduced significantly compared to untreated animals. The disappearance of the photoacoustic signal was associated with the death of the adipocytes treated with hyaluronate hollow AuNSs. Although the study might have been terminated after 2 h of treatment (authors did not specify), there is clear evidence that these NPs can be used to induce PTT for reduction of WAT mass and reversal of obesity. It would have been interesting to follow up on the animal body weight after treatment. Nonetheless, the study presents an opportunity for the non-invasive transdermal delivery of PTT agents in the WATs [18].

An independent study reported on the effects of NanoLipo, a AuNR solution injected on the abdominal tissue of Yucatan mini pigs. When exposed to NIR laser, NanoLipo was able to uniformly and selectively heat up the WATs while sparing the surrounding tissues. The AuNR solution used in this study absorbs light at 800–900 nm which is within the tissue transparency window, while the endogenous chromophores (water and hemoglobin) have lower absorption and the FDA-approved laser-assisted liposuction is at ≥ 1000 nm. Laser-assisted liposuction involves insertion of laser probes into the subcutaneous tissue to liquefy small volumes of fat because water absorbs and emits heat at the same wavelength. As a result the surrounding tissues, including muscles and fibrous connective tissues, are also affected by the heat. NanoLipo uses external laser which only affects the abdominal tissues in which the AuNRs are localized, this enhances the selectivity and safety of the treatment. The procedure was well tolerated, swelling healed faster, the NRs are easily aspirated as they do not bind to the WATs, and the AuNR dosage used (0.01–0.05 g/kg body weight) is below the expected toxicity limit (3.2 g/kg) for gold. NanoLipo was able to remove double the fat content within a short period when compared to the conventional suction-assisted lipectomy [19].

## Biomedical application of nanotechnology

Nanotechnology has revolutionized the field of medicine, with the promise of safer and more selective drug formulations. NPs have unique properties that can be manipulated for biological applications, due to their small size and large surface area, they can be used for multimodal applications [58, 60, 62, 104]. These properties allow for attachment of multiple molecules on a single particle, creating biocompatible multimodal systems for diagnosis and treatment of diseases. The nanocarriers (liposome, metallic NPs, polymeric NPs) can be easily modified with targeting molecules (such as peptides, antibodies, aptamers, etc.) to achieve specificity and selectivity, and therapeutic molecules (drugs) for targeted disease treatment [59, 60], as well as imaging agents to monitor the disease response to treatment in real time [58]. By manipulating the NP’s physico-chemical properties (size, shape and surface chemistry), the interactions of the NPs with the biological systems, biodistribution and their clearance by the RES can be controlled [105]. A change in these parameters can significantly influence the pharmacokinetics of the nanoformulations, circumvent drug limitations by increasing drug stability, circulation time, prevent biodegradation, and control drug release. NP size is fundamental in determining the rate at which NPs are cleared by the body’s RES organs and mononuclear phagocyte system. Size also facilitate NP uptake by penetrating highly impermeable cellular barriers; thus, improving the delivery and efficacy of the drug to its specific target [106].

### Nano-based drug formulations in clinical practice

Nanomedicine refers to the application of nanoscience and nanotechnology in medical sciences, for treatment, diagnosis and imaging purposes [58]. A number of nano-based formulations are either available for human use as FDA-approved medicines, or undergoing different phases of human clinical trials [58–60, 107]. By 2016, already 51 FDA-approved nanotherapies were available while 77 nanoproducts were in clinical trials [107]. Majority of these nanoproducts contained drugs previously approved by the FDA. In principle many of the nanodrugs are designed as encapsulations of FDA approved drugs in biodegradable and biocompatible NPs such as liposomes, micelles or polymeric NPs [58–60, 107]. Inorganic or metallic nanomaterials such as AuNPs, iron oxide, silica etc., are still in phase I–III clinical trials [59, 107, 108]. The lack of information on the fate of metallic NPs in vivo, could be the reason why there is a delay in their translation and transition into clinical medication. Metallic NPs are non-biodegradable in nature; their ability to pass through cellular components raises health concerns.

Various nanocarriers used in clinical practice serve as drug delivery agents to ascertain distribution of drugs/treatment to desired targets [108]. Most of the nanoformulations in clinical trials are untargeted and rely on the EPR effect to target and accumulate in the diseased tissues [108]. In contrast, targeted therapies use ligands that bind to disease associated biomarkers and accumulate specifically in cells that express the target receptor. Both the passive and active-targeted nanosystems demonstrated reduced bystander effects and enhanced the drug’s therapeutic index in pre-clinical and clinical studies [58–60].

Nanomedicines have been on the market since 1990 for treatment of various diseases including cancer, severe combined immunodeficiency disease, and arthritis [59, 60, 107]. Most of the nanoformulations are comprised of drugs currently used clinically for disease treatment. The drugs are encapsulated within the nanomaterials to improve their pharmacokinetic properties [107]. Some of the nanodrugs already on the market are listed in Table 2. The earliest application of nanomedicine was in cancer treatment, where liposomes were used to deliver chemotherapeutic payloads to the tumor site [109]. Doxil and Abraxane are the first FDA-approved chemotherapeutic nanodrugs in 1995 and 2005, respectively. Abraxane, an albumin-bound paclitaxel NP formulation, was approved for treatment of metastatic breast cancer in the United States of America (USA) and showed greater efficacy with an improved safety profile than free paclitaxel [110, 111]. Doxorubicin is one of the drugs used for treatment of cancer and is associated with cardiac toxic side effects. The biodegradable liposomes used to deliver Doxorubicin to the tumor, retains the drug efficacy and increase circulation time compared to the free drug. Doxil is FDA-approved for treatment of refractory ovarian cancer, breast cancer and Kaposi’s sarcoma [111, 112]. The number of diseases treated by Doxil has since increased, covering a wider range of diseases including multiple sclerosis, neutropenia, anemia (refer to Table 2). The nanosystems had reduced systemic toxicity and enhanced tumor specificity. Some of the nanodrugs rely on the EPR effect to target diseased cells/tissues, especially in cancer [107]. In a pathological state, EPR is characterized by pathological and excessive angiogenesis and increased secretion of various permeability mediators that provide an opportunity for more selective targeting of NPs as it does not occur in normal tissues or organs [6, 54, 55]. These effects can be further enhanced by targeting disease-specific markers, active targeting can be achieved by using antibodies peptides, aptamers or ligands that binds to these disease biomarkers with high affinity [11].

**Table 2:** FDA-approved nanodrugs in the market

Nanodrug tradename	Nanocarrier	Drug	Application			Company
Abraxane	Albumin NPs	Paclitaxel	Breast cancer, metastatic pancreatic cancer, non-small cell lung cancer			Celgene
Albelcet	Liposomes	Amphotericin B	Fungal infection			Sigma-Tau
Curosurf		Poractant alfa	Respiratory distress syndrome			Chiesi USA
Doxil		Doxorubicin	Karposi’s sarcoma, ovarian cancer, multiple myeloma			Janssen
DepoDur		Morphine sulphate	Postoperative analgesia			Pacira Pharmaceuticals
Marqibo		Vincristine sulfate	Acute lymphoid leukemia			Talon Therapeutics
Thermodox		Doxorubicin	Hepatobiliary tumors			Celsion corp
Copaxone	Polymeric NPs	Glatiramer acetate	Multiple sclerosis			Teva Pharmaceuticals
Neulasta		Pegfilgrastim	Neutropenia			Amgen
Paclical	Micelle NPS	Paclitaxel	Ovarian cancer			Oasmia Pharmaceutical
Rapamune	Nanocrystals	Sirolimus	Lymphangioleiomyomatosis and an immunosuppressants			Elan Nanosystems (now Alkermes)
Emend		Aprepitant	Prevent nausea and vomiting after surgery or chemotherapy			Merck
Tricor		Fenofibrate	Reduces cholesterol and TAGs			AbbVie
Feraheme	Inorganic NPs	Ferumoxytol	Anemia associated with chronic kidney disease			AMAG Pharmaceuticals

### Potential clinical applications of nanodrugs in obesity

There are currently no nano-based drugs that are either clinically approved or in clinical trials for the treatment of obesity. However, based on published literature reviewed here and the potential of the nanodrugs, it is just a matter of time before these nanodrugs will be tested in clinical trials and made available to the market. The studies reviewed here provide definitive proof of the concept that nanomedicine can be a feasible strategy for the treatment of obesity, by improving patient’s compliance and possibly eradicate most of the challenges associated with the commercially available anti-obesity drugs [12–15]. The nanodrugs listed in Table 2 have been used successfully in treatment of chronic diseases, especially cancer. Since cancer and obesity share some similar characteristics (e.g. high proliferation rate, compromised vascular system angiogenesis) [6, 37, 54, 55], nanomedicine can work quite as good in obesity. So far, since obesity has benefitted from similar treatment strategies as cancer, it might also benefit from nano-based anti-angiogenic, anti-neoplastic [37] and transdermal therapies in future [18, 19].

The reviewed strategies prove that it is possible to reduce body weight by targeting the WAT vasculature using nano-based anti-angiogenic inhibitors [13, 14], WAT browning agents [12, 16], and photothermal agents [18, 19]. The three strategies were effective, not only for reversal of obesity at lower doses, but also for reduction of bystander effects and normalization of metabolic activities. More importantly, these nanosystems delivered non-specific and poorly soluble drugs to the target and confined their effects directly on the diseased cells while sparing surrounding tissues [12, 74]. Non-invasive and painless microneedle patches also presented a promising platform for transcutaneous anti-obesity drug delivery systems [16].

The ability of nanocarriers to increase the bioavailability of drugs was demonstrated by using some drugs with anti-obesity effects, current and those that were withdrawn. Encapsulating cannabinoids in NPs increased transportation across the hydrophobic mucosa, reduced adverse effects [113], enhanced drug absorption in the intestinal tissues and drug biodistribution in rat brains [114]. Nano-based delivery systems further improved the stability and activity of orlistat, where the nanoemulsified orlistat showed enhanced dissolution rate and lipase inhibition action when compared to the free drug [115]. Similar effects were observed when orlistat was embedded in nanoemulsified multi-unit pellet system [116], and polymer nanocarriers [117]. These systems were able to mask the drug’s hydrophobicity, reduced orlistat induced side effects and increase its bioavailability [115–117]. The efficacy and bioavailability of rimonabant, an anti-obesity drug withdrawn due to lethal effects, was improved by encapsulating the drug in nanostructured lipid carriers [118] and amorphous mesoporous magnesium carbonate [119]. Although the therapeutic effects of orlistat and rimonabant nano-drugs is yet to be evaluated in vivo [118], the nano-based formulations represent an important tool for improvement of existing and withdrawn drugs. Anti-obesity drugs such as rimonabant and sibutramine can thus be repurposed using the nanotechnology-based delivery systems to reduce toxic effects and increase drug efficacy.

## Conclusions

Obesity is a global health threat and requires urgent attention to reverse the disease and prevent its progression to other chronic diseases [4, 5]. The conventional therapies, which are only successful for a limited period, brings with them undesirable bystander toxic effects [5]. Nanotechnology-based strategies have shown potential in improving drug delivery, drug uptake [15, 63] and therapeutic index of potential anti-obesity agents in pre-clinical studies [13, 14, 62]. Furthermore, nanotechnology also improved the pharmacokinetics of anti-obesity drugs (current and withdrawn) [115–119]. The FDA-approved nanodrugs showed promising clinical health benefits in cancer treatment for over 20 years [58, 59], and based on Sect. 4.2, they could significantly improve the safety and efficacy of the anti-obesity drugs [114–120]. Nano-based delivery systems offer great opportunities to rescue anti-obesity drugs that were withdrawn from the market [114–120], by enhancing their sensitivity and selectivity through targeting of disease-associated biomarkers [13–15]. These nanosystems can be useful in addressing drawbacks associated with anti-obesity drugs, especially non-specificity, use of large doses, and poor drug solubility [114–120]. The reviewed preclinical studies proved that targeted nanotherapy can be delivered to diseased WATs and minimized off-target toxic effects [13, 14]. Targeting the WATs and its vasculature through nano-based systems could be an ideal target in developing therapeutic strategies with sustainable anti-obesity effects.

Orally administered drugs such as orlistat suffer from poor solubility [31, 32], therefore drug specificity and efficiency can be improved by using nano-based systems. The delivery systems can be tailored based on the drug’s properties, and exploiting the NP’s physico-chemical properties. Concern about metal NP toxicity can also be addressed by using biodegradable nanocarriers such as liposomes and polymers which are already used clinically due to their biocompatibility [59, 60]. These systems, especially liposomes have been used for > 20 years as drug delivery systems [120]. Drug release is easily controlled, thereby increasing drug specificity by creating systems that are responsive to the disease environment, such as pH and temperature. This review highlights the nano-based strategies for the improvement in the reversal of obesity and obesity-induced diseases, and can help curb the epidemic in its fracks and reduce obesity-associated chronic diseases.

## Data Availability

Not applicable.
